# A Safe and Efficient Brain–Computer Interface Using Moving Object Trajectories and LED-Controlled Activation

**DOI:** 10.3390/mi16030340

**Published:** 2025-03-16

**Authors:** Sefa Aydin, Mesut Melek, Levent Gökrem

**Affiliations:** 1 Department of Electronics and Automation, Gumushane University, 29100 Gumushane, Turkey; mesutmelek@gumushane.edu.tr; 2 Department of Electrical and Electronics Engineering, Tokat Gaziosmanpasa University, 60600 Tokat, Turkey; levent.gokrem@gop.edu.tr

**Keywords:** electroencephalography, electrooculography, brain–computer interface, machine learning, classification, Emotiv Epoc

## Abstract

Nowadays, brain–computer interface (BCI) systems are frequently used to connect individuals who have lost their mobility with the outside world. These BCI systems enable individuals to control external devices using brain signals. However, these systems have certain disadvantages for users. This paper proposes a novel approach to minimize the disadvantages of visual stimuli on the eye health of system users in BCI systems employing visual evoked potential (VEP) and P300 methods. The approach employs moving objects with different trajectories instead of visual stimuli. It uses a light-emitting diode (LED) with a frequency of 7 Hz as a condition for the BCI system to be active. The LED is assigned to the system to prevent it from being triggered by any involuntary or independent eye movements of the user. Thus, the system user will be able to use a safe BCI system with a single visual stimulus that blinks on the side without needing to focus on any visual stimulus through moving balls. Data were recorded in two phases: when the LED was on and when the LED was off. The recorded data were processed using a Butterworth filter and the power spectral density (PSD) method. In the first classification phase, which was performed for the system to detect the LED in the background, the highest accuracy rate of 99.57% was achieved with the random forest (RF) classification algorithm. In the second classification phase, which involves classifying moving objects within the proposed approach, the highest accuracy rate of 97.89% and an information transfer rate (ITR) value of 36.75 (bits/min) were achieved using the RF classifier.

## 1. Introduction

Nowadays, many serious health problems negatively affect people’s quality of life. One of these problems is stroke, which results in a person losing their ability to move and becoming bedridden. Although individuals with this condition have normal brain activity, they are confined to bed due to neurological disorders and have difficulty meeting their daily needs. Brain–computer interface (BCI) systems have been developed to assist these individuals in fulfilling their basic daily needs. BCI systems are designed to produce meaningful outputs by measuring the potential differences in the human brain. Neurons located on the human brain’s surface constantly interact with each other. The rhythms resulting from these interactions are grouped according to their frequency values. These groups are known as alpha, beta, theta, delta, and gamma waves. Table 1 provides the frequency and amplitude values of these waves.

The delta wave, which has a frequency range of 0.5–4 Hz, is the slowest wave with the highest amplitude (Table 1). This type of wave is seen in infants up to one year old and in adults during deep sleep [1]. Theta waves, ranging from 4–7 Hz, are observed during light sleep and relaxation. Alpha waves, appearing in the 7–12 Hz range, are associated with eye closure and relaxation. Beta waves, observed in the 12–30 Hz range, dominate during states of alertness and anxiety. These waves are also elevated in individuals solving mathematical problems [2]. Gamma waves, with frequencies above 30 Hz, have the lowest amplitude and play a crucial role in detecting neurological diseases. They relate to perception, recognition, and similar cognitive functions [3]. These waves, categorized by their frequency values, are frequently used in BCI systems.

**Figure 1:**
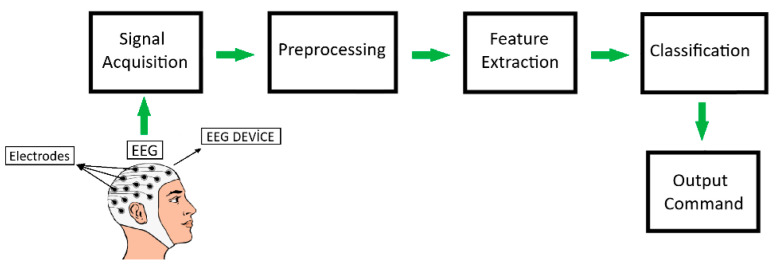
The basic scheme of BCI systems.

BCI systems typically use electroencephalography (EEG), a non-invasive method that measures a person’s brain signals without requiring surgical intervention. In this method, the signals measured from the individual’s brain activity are processed and taught to machines. Associating the output obtained from signal processing with a command allows for controlling electronic devices such as electric wheelchairs, beds, and lamps [4]. This function enables individuals with paralysis to interact with their environment and meet their needs. The basic scheme used in BCI systems is depicted in Figure 1.

As shown in Figure 1, BCI systems are fundamentally composed of four groups. These groups are signal acquisition, preprocessing, feature extraction, and classification stages. In the signal acquisition stage, brain signals are recorded using EEG devices. During the preprocessing stage, operations such as trimming, filtering, and normalization are applied to the recorded data. Feature extraction methods are applied to the filtered data. The customized data are then classified in the classification stage, the final step of signal processing, using different learning algorithms. Then, an output command is generated based on the values obtained from classification.

In EEG-based BCI systems, methods such as visual evoked potential (VEP) [5], motor imagery (MI) [6], and P300 [7] are frequently used during the signal acquisition phase. The P300 method involves a positive deflection in brain waves in response to certain stimuli such as lights, sounds, or various visual cues. P300 waves exhibit a positive deflection within a 300 ms to 600 ms time window and are recorded using EEG devices. In the MI method, the physical movements of the individual are replaced by imagined mental movements. When a person imagines any physical movement, specific patterns emerge in the brain, which are recorded using EEG devices. The recorded EEG signals from all these methods are processed using signal-processing techniques in a computer environment. Output is generated through classification based on the detected value from signal processing. In the VEP method, visual stimuli such as flashing lights or images at different frequency values are presented to the subject, and the resulting voltage changes in the brain are recorded. In the VEP method, when the frequency value exceeds 6 Hz, a state known as steady-state visual evoked potential (SSVEP) occurs.

An SSVEP-based BCI system consists of three main components: data acquisition, signal processing, and output command. In this system, visual stimuli with different frequency values are shown to the subject, and SSVEPs are stimulated in the brain [8]. Potential changes are recorded through EEG devices. The created dataset is processed through computers and converted into command outputs. The resulting output commands are used to control peripheral devices. SSVEP stands out with its non-invasive structure, high ITR and signal-to-noise ratio (SNR) values, short training time, low training data, and low mental workload of the user [9]. SSVEP responses, which can be seen in the 1–100 Hz range, are stronger at frequency values below 15 Hz [10]. To evaluate the performance of an SSVEP-based BCI, first the SSVEP response is converted into the frequency domain and visualized using SNR. Then, the system’s classification accuracy, response time, and number of recognized targets are determined. Classification accuracy is largely affected by the strength and SNR value of the SSVEP response, while speed is related to the time required for the SSVEP signal to reach sufficient power. The number of targets, which determines the command options the system can offer, can have a direct impact on both accuracy and speed [11].

Compared to P300 and MI methods, SSVEP-based BCI systems stand out with the advantages of short training time and high accuracy rate and ITR value. While MI-based BCI systems require users to mentally visualize hand, foot, etc., movements and go through long-term training processes, the SSVEP method is based on natural responses produced by the brain to direct visual stimuli. This makes the learning process of SSVEP-based systems significantly shorter and their use more practical. Additionally, SSVEP-based systems allow the simultaneous presentation of multiple visual stimuli at different frequencies. This feature eliminates the need for sequential scanning methods commonly used in P300-based BCI systems, making the multiple target selection process faster and more efficient. Visual stimuli directly creating a response in the occipital cortex enables the system to achieve high SNR and thus increases the reliability of signal detection. SSVEP-based BCI systems offer an accessible and effective alternative for a wide range of users with their advantages. They stand out as an important communication and control tool, especially for paralyzed individuals or patients with loss of motor control, thanks to their user-friendly interface and reliable performance [12,13]. When the studies are examined [14,15,16,17,18], it is seen that SSVEP-based systems are widely used in hybrid form with other signals such as electromyography (EMG) and electrooculography (EOG) due to their advantages.

The primary goal of developing EEG-based BCI systems is to benefit individuals who have lost their ability to move. However, while these systems offer advantages, they also have some disadvantages. These issues can be listed as follows:

➢ Research and testing of the systems are only conducted in laboratory environments;
➢ The comfort of these systems, which users must rely on for life, is insufficient;
➢ The systems operate slowly;
➢ They are generally high cost, which limits their accessibility to a broader audience;
➢ The systems are not particularly suited for long-term use by the user [19].

In addition to these disadvantages, EEG devices are relatively cost-effective compared to recording techniques such as magnetoencephalography (MEG) and functional magnetic resonance imaging (fMRI). However, they remain prohibitively expensive for daily use by individuals with disabilities [7]. Non-medical EEG devices produced for EEG-based BCI systems research are slower than medical-grade EEG systems. For example, the low-cost Emotiv Epoc X EEG device produces lower-quality signals and operates at a lower speed compared to G.Tec, a medical-grade EEG system [20,21,22,23]. In a study comparing the synchronization of two devices, a delay of 162.69 ms was calculated for Emotiv EPOC, while a delay of 51.22 ms was calculated for G.Tec [24]. Another problem is that the visual and mental fatigue caused by vibrating stimuli in visual stimulus-based BCI systems hinders users’ adoption of such systems and complicates their usage [25]. Furthermore, users must continuously focus on visual stimuli for the system to be active. Nevertheless, research has shown that users experience eye problems over time due to prolonged and continuous exposure to visual stimuli [26]. This serious issue impedes users’ long-term use of these systems and threatens eye health. Additionally, in visual stimulus-based systems, the frequency values of the stimuli create potentials in the brain via the eyes, rendering these systems unsuitable for individuals with preexisting eye health issues.

In this study, a new hybrid system that uses EOG artifacts contained in EEG signals and the SSVEP method was developed in order to provide an alternative to the eye health problems caused by the visual stimuli contained in visual stimulus-based BCI systems in system users and to increase the usage time, comfort, usability, and safety of the system. The SSVEP method is used more frequently than other common BCI paradigms because it can be more easily detected in EEG signals and does not require the creation of any training set [27]. Therefore, in the current study, the SSVEP method was preferred to activate the system. Additionally, synchronized operation of BCI systems is a known challenge of these systems [28].

In EEG signals recorded with an EEG device, EOG artifacts occur as a result of eye movements, mostly in the channels located in the frontal lobe of the brain. Artifacts are unwanted signals that can negatively impact neurological processes. These signals may influence the characteristics of neurological events and may even be mistakenly perceived as control signals in BCI systems [29]. EOG artifacts are filtered through various methods when they are not intended to affect neurological conditions. However, in studies where eye movements need to be detected [30], EOG artifacts found in EEG signals can be used as a source. Particular eye movements, including blinks, upward and downward glances, leftward and rightward shifts, and eye closures, can be identified, isolated, and categorized using EEG data. These detected movements can subsequently be linked to distinct command outputs for use in a BCI typing system [31].

**Figure 2:**
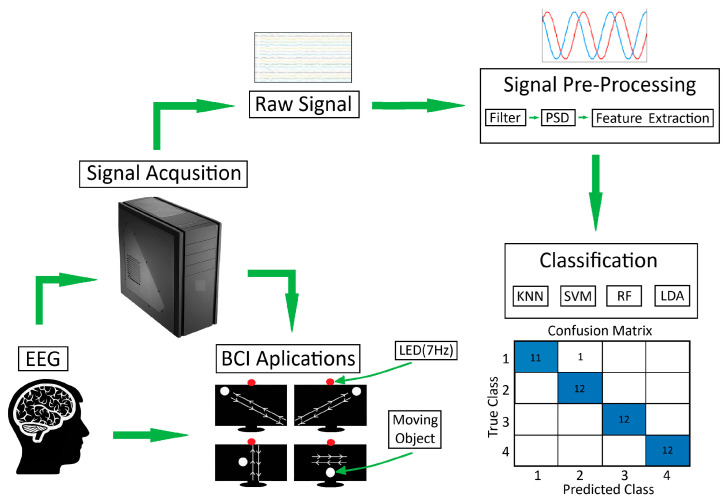
General schematic of the designed BCI system.

The hybrid system designed in the study proposes an innovative approach of moving objects (white balls), each with different routes, instead of the visual stimuli contained in visual stimulus-based BCI systems. In the system, moving balls are shown to the user via a computer screen. The command generated, which depends on the output assigned to the orbital movement focused on by the user, is sent as a control signal to peripheral devices (bed, wheelchair, etc.). However, this proposed approach has a drawback. The system user may involuntarily or unconsciously make the same orbital movement with the moving balls without looking at the monitor, and the system may be activated involuntarily. In order to solve this problem, a light-emitting diode (LED) with a frequency of 7 Hz was placed in the upper middle of the screen as a condition for the system to be active. When activation of the system with conscious eye movements is wanted, the system checks for the presence of the LED at the point of view through the SSVEP method and is not activated in cases where the LED cannot be detected. Thus, it uses the LED as the safety valve. The designed BCI system is shown in Figure 2.

In the system, the general scheme of which is given in Figure 2, since the number of LEDs is 1, not much light is emitted. In addition, the system does not require the user to look directly at or focus on the visual stimulus. In common SSVEP-based BCI systems, candidates are required to focus and look at least four flashing lights [32]. In P300-based systems, it is necessary to look at the light at least two or more times for each command [33]. However, in the proposed system, the candidate only needs to look in the direction of a flashing visual stimulus without focusing. Thus, the system user can use the system safely through a single visual stimulus, without the feeling of glare in the eye and without being exposed to the disturbing effect of light.

The designed system uses the 14-channel Emotiv Epoc X (EMOTIV Inc., San Francisco, CA, USA) EEG device, which is cost-effective, portable, wireless, and easy to clean and use. Data in the study were recorded at a sampling frequency of 256 Hz. The recording process was done in two stages: with the light on (illuminated) and with the light off (non-illuminated). Pre-processing steps such as cutting and filtering were applied to the recorded data, features were extracted, and effective channel selection was made. The features extracted using active channels were classified in two stages. In the first classification stage, the data recorded with the light on and off were distinguished from each other using the trapezoidal method with the SSVEP method. In the second classification stage, EEG data containing EOG artifacts detected in the LED active state in the background in the first stage were classified in order to distinguish objects moving up-down, left-right, right-cross, and left-cross.

This study is aimed to find solutions to the feeling of glare in the user’s eyes and eye health problems that occur over time, caused by the visual stimuli contained in visual stimulus-based BCI systems. Since these problems directly negatively affect the system usage time, usability, and comfort, a more usable, safe, and comfortable system is designed with the proposed hybrid method and approach. In the study, EOG artifacts contained in EEG data were recorded through moving objects suggested in the approach, while the 7 Hz LED, which is the condition for the system to be active, was active and disabled. Active channel selection was made through the recorded signals, and moving objects were classified with machine learning algorithms using EOG artifacts of the detected active channels. The detection of the LED, which acts as a safety valve, was made through the trapezoidal method using the SSVEP method. The proposed study is a hybrid system, as it uses EOG artifacts in EEG signals to classify moving objects and uses the SSVEP method to detect the presence of LED by the system. The study proved that the LED placed in the background can be detected without the need for focusing and that moving objects can be classified using the EOG artifacts in the EEG signals through the proposed approach. Thus, a hybrid BCI system that is relatively harmless to visual stimulus-based BCI systems in terms of eye health, relatively more comfortable, suitable for long-term use, and safer in terms of control has been proposed.

## 2. Related Works

BCI is a direct interaction, communication, and control system established between the brain and external devices [34]. SSVEP and P300 visual stimulus-based paradigms are frequently used in BCI systems. SSVEP is a classical BCI paradigm that has been extensively studied for more than 20 years [35] due to its advantages. SSVEP has a higher SNR value and requires less training than other methods such as P300 and MI [36]. Since the method does not require any mental effort, as in the MI method, it is easier to apply and less tiring [37]. Studies have shown that the method gives relatively higher accuracy and ITR values than other methods [38]. It can also be detected more easily in EEG signals [27]. However, SSVEP-based BCI systems cause eye fatigue and a feeling of glare in users due to the visual stimuli they contain [39]. This significantly reduces the usage time of the systems and negatively affects system comfort. Another important disadvantage of the systems is their high cost [40]. In order to provide solutions to existing problems, a hybrid system that uses EOG artifacts occurring in SSVEP and EEG signals is proposed in the study. The proposed system aims to free the user from visual stimuli through the moving objects approach. As a condition for the system to be active, 1 LED at 7 Hz was used. In addition, the Emotiv Epoc X (EMOTIV Inc., San Francisco, CA, USA) wearable EEG device [41] was preferred in the designed system due to its cheap cost, long battery life, short installation time, and ease of use.

In this section, a literature review was conducted on hybrid BCI systems in which SSVEP and EOG signals are used together and SSVEP-based BCI systems using the Emotiv Epoc EEG device.

In one study, researchers [14] proposed a hybrid BCI system by combining the SSVEP method and EOG signals. In the study, researchers obtained the prior probability distribution of the target with the SSVEP method in order to reduce the transition time between tasks of the SSVEP-based system. They obtained target prediction output by using EOG signals to optimize the probability distribution. In another study [30] where EOG signals and SSVEP signals were used together, researchers controlled a robotic arm with six degrees of freedom. Designing an EOG-based switch using triple eye blink, the researchers achieved an accuracy rate of 92.09% and an ITR value of 35.98 (bits/min) in the experiment. In another study [15], researchers proposed a new hybrid asynchronous BCI system based on a combination of SSVEP and EOG signals. The researchers used 12 buttons, each representing 12 characters with different frequency values, to trigger SSVEPs in the interface they designed. They recorded signals on 10 subjects by changing the size of the buttons. At the end of the study, they stated that the asynchronous hybrid BCI system has great potential for communication and control. In another study [16] where SSVEP method and EOG signals were used in the same system, researchers used 20 buttons corresponding to 20 characters in the interface they designed. Researchers used ten healthy subjects in the study and asked the subject to look at the lights, each of which was flashing at the same time during the experiment. They recorded EOG signals when the buttons moved in different directions. At the end of the experiments, they achieved an accuracy rate of 94.75%. In another study [17], which combined the SSVEP method and EOG signals, a hybrid printer system was developed using 36 targets. The researchers divided the targets into nine groups of letters, numbers, and characters. While using EOG signals to detect the target group, they identified the target within the selected group with SSVEP. Researchers tested the proposed system on ten subjects and obtained an accuracy rate of 94.16%. In a different study that [18] used both methods together, researchers presented a comparison dataset for BCI systems. The dataset consisted of data from the SSVEP-based BCI system and the SSVEP-EMG and SSVEP-EOG-based BCI systems. They conducted the experiments using a virtual keyboard containing nine visual stimuli flashing between 5.58 Hz and 11.1 Hz. They used ten participants in the copywriting task and collected data for 10 sessions for each system. They evaluated the systems with criteria such as accuracy, ITR, and NASA-TLX workload index.

In a visual stimulus-based BCI system [42], researchers designed a screen containing visual stimuli at frequencies of 7 Hz, 9 Hz, 11 Hz, and 13 Hz. They recorded signals over 10 min sessions. To augment the dataset, white noise with amplitudes of 0.5 and 5 was added to augment the size of the training set threefold. The classification was performed using support vector machine (SVM) and k-nearest neighbors (k-NN) classifiers. Without data augmentation, they achieved 51% and 54% accuracy rates, respectively. The accuracy rates improved with augmented data to 55% and 58%. In another study [43], a drone controlled by EEG signals was developed and tested on 10 healthy subjects and detected visual stimuli at frequencies of 5.3 Hz, 7 Hz, 9.4 Hz, and 13.5 Hz. This system resulted in an average accuracy of 92.5% and an ITR value of 10 bits/min in the BCI system. In another study [44], four LEDs with frequencies of 13 Hz, 14 Hz, 15 Hz, and 16 Hz were placed outside a visual interface. Next, the system was tested on five participants who completed image flickering experiments at four different frequencies in four directions. In this research, 23 participants were asked to complete tasks in different rooms. However, 12 participants either could not complete the tasks or did not achieve sufficient results. Those who completed all three tasks obtained an average accuracy of 79.2% and an ITR of 15.23 (bits/min). These studies indicate that systems incorporating multiple visual stimuli with different frequency values pose risks to users’ eye health due to the need for sustained focus. Consequently, these systems are uncomfortable and unsuitable for prolonged use. In this respect, some studies [44] have observed that users experienced visual stimulus-related issues during the experiment, leading to task failure. Besides, the signal recording durations in these studies are often long, and the ITR values of the systems are relatively low.

Various studies have been conducted in the literature to mitigate the negative effects of visual stimuli on system users. For instance, in [45], a BCI system operating at high frequencies (56–70 Hz) was proposed to reduce the sensation of flicker caused by vibrating stimuli. The system was tested with low-frequency (26–40 Hz) stimuli. The study achieved accuracy rates of 98% for low-frequency stimuli and 87.19% for high-frequency stimuli. The ITR of the system was calculated to be 29.64 (bits/min). This study demonstrated that while accuracy rates decreased with higher frequency values, the system did not provide a solution to the negative effects of visual stimuli on users. In another study [46], a BCI system based on a rotating wing structure was proposed, wherein five healthy subjects aged between 27 and 32 participated. The designed interface had a black screen divided into four sections, each featuring a wing with an “A” mark. Each wing completed its rotation at different speeds and directions. Using the cubic SVM method, the researchers recorded data for 125 s per class and achieved the highest success rate of 93.72%. A review of these studies indicates that the results obtained by these systems do not provide a permanent solution to the existing problems. Moreover, these systems have long recording times, relatively low accuracy rates, and vulnerability to unintended eye movements.

Overall, relevant studies have focused on eye movements for BCI systems as an alternative to visual stimuli. For instance, in [47], eye movements were used to control a wheelchair. This research proposed a brain activity paradigm based on imagined tasks, including closing the eyes for alpha responses and focusing attention on upward, rightward, and leftward eye movements. The experiment was conducted with twelve volunteers to collect EEG signals. Employing a traditional MI paradigm, the researchers achieved an average accuracy of 83.7% for left and right commands. Another study [48] examined the relationship between eye blinking activity and the human brain. This research used channels AF3 and F7 for the left eye and AF4 and F8 for the right eye. Through a convolutional neural networks (CNN) structure in a different study focusing on eye movements [49], researchers investigated the effect of visual stimuli on the classification accuracy of human brain signals. The study involved 16 healthy participants who were shown arrows indicating right and left directions while their brain beta waves were recorded. Using SVM methods, the researchers achieved an average accuracy of 70% in standard tests and an accuracy of 76% in tests with effective visual stimuli. These studies primarily focus on the eye movements of the system user. However, systems remain vulnerable to unintended eye movements made by the user. Implementing sequential movement coding makes it challenging for users to adapt to the system, resulting in increased error rates. Additionally, these systems often have long recording times and relatively low accuracy rates.

The P300 method is among the frequently used techniques in the field. Using this method, researchers [50] classified P300 signals obtained from six healthy subjects aged 20 to 37 using deep learning (DL) techniques. The interface they designed required participants to follow two different scenarios. For classification, they employed a five-layer CNN deep learning module. The results showed that with deep learning classification, the transition time between stimuli achieved 100% success in training data. Meanwhile, in test data, 80% success was achieved with a 125 ms inter-stimulus interval and 40% success with a 250 ms inter-stimulus interval. In another study using the P300 method [51], researchers developed a hybrid BCI hardware platform incorporating both SSVEP and P300. They created a chipboard platform with four independent radial green visual stimuli at frequencies of 7 Hz, 8 Hz, 9 Hz, and 10 Hz. The platform was designed to extract SSVEP and four high-power red LEDs flashing at random intervals to evoke P300 events. The platform was tested with five healthy subjects. The researchers successfully detected P300 events concurrently with four event markers in the EEG signals. In another study [52], researchers investigated pattern recognition using the P300 component induced by visual stimuli. The study involved 19 healthy participants. Participants were instructed to look at a screen and count how many times it flashed, and the data were recorded. The researchers found that Bayesian networks (BN) achieved the highest accuracy rate of 99.86%. These studies indicate that while visual stimuli are actively used in these methods, the systems often do not provide sufficient comfort for the user.

In [53], the MI method was used to examine the control of a spider robot. The researchers recorded EEG signals and tested the detection of imagined hand movements for controlling the robot. Specifically, the imagined opening of the hand was associated with forward movement of the robot, while the imagined closing of the hand was associated with backward movement. Through a CNN, the researchers achieved a maximum classification accuracy rate of 87.6%. In another study on MI [54], researchers analyzed MI data obtained over 20 days from a participant. The study involved commands for right, left, up, and down movements. During the classification phase, they employed the ensemble subspace discriminant classifier and achieved an optimal daily average accuracy of 61.44% for the four-class classification. For five participants, the average accuracy for the four-class classification was 50.42%, while the binary classification accuracy for right and left movements was 71.84%.

In [55], the control of a wheelchair was investigated. To this end, three white LEDs with frequencies of 8 Hz, 9 Hz, and 10 Hz were installed in the screen’s right, left, and upper corners, respectively. The setup was tested on five different participants aged between 29 and 58 years, with movements including left, right, and forward. These authors employed canonical correlation analysis (CCA) and multivariate synchronization index (MSI) methods to determine the dominant frequency. The researchers achieved an accuracy rate of 96% for both methods. In [56], the authors differentiated error-related potentials (ERP) in both online and offline conditions with 14 participants in a visual feedback task. Participants were shown red, blue, and green visual stimuli at periods of 500 ms, 700 ms, and 3000 ms. The results showed an accuracy rate of 81% using deep learning techniques. In another study [57], a system was designed to detect emotional parameters such as excitement, stress, focus, relaxation, and interest. Participants were shown a 15 min mathematics competition video to evoke excitement, attention, and focus. The researchers tested the collected experimental datasets using naive Bayes and linear regression learning algorithms. The linear regression classifier achieved an accuracy rate of 62%, while the naive Bayes classifier achieved an accuracy rate of 69%.

In a study investigating the impact of adjustable visual stimulus intensity, researchers [58] designed a system to examine the effects of LED brightness on evoking SSVEP in the brain. The LED frequencies were set to 7 Hz, 8 Hz, 9 Hz, and 10 Hz; the brightness levels were adjusted to 25%, 50%, 75%, and 100%; and the system was tested on five individuals. The study found that the highest median response was achieved with a brightness level of 75%, which provided the highest SSVEP responses for all five participants. However, the 75% brightness level, despite yielding the best response, was found to be uncomfortably high for system users. Additionally, the number of visual stimuli used in the system was quite large. Thus, the applied method and obtained results do not provide an effective solution.

## 3. Materials and Method

### 3.1. EEG Device

**Figure 3:**
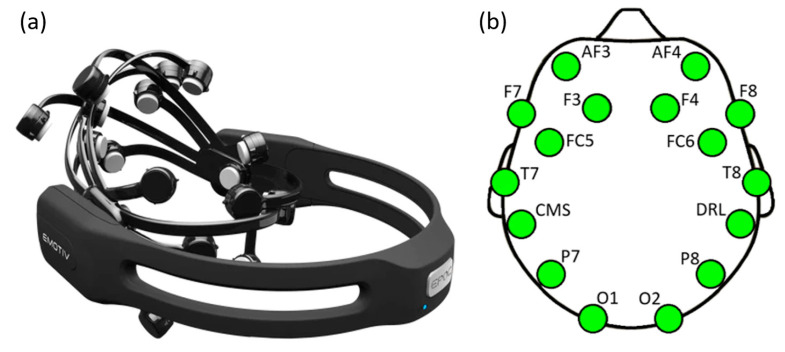
Emotiv Epoc X EEG device (**a**) and positions of the electrodes on the head (**b**).

When designing BCI systems, the goal is to select a wearable, wireless, low-cost, and high-comfort EEG device for data recording. In addition, factors such as the number of channels, cost, setup time, ease of use, and the type of BCI application play a crucial role in selecting the EEG device [59]. The Emotiv Epoc X device has 14 channels. This system is powered by lithium batteries, providing active mobile use for up to approximately 12 h. The device’s wireless capability and adjustability to head sizes facilitate its use. Its affordability, setup, and installation time of approximately 15 min also provide advantages for BCI systems. The EEG device and positions of the electrodes on the head are shown in Figure 3.

The Emotiv Epoc X EEG device, shown in Figure 3a, uses a special saline (salt water) solution to reduce the impedance between the electrodes and the scalp. Using the saline solution means the participant does not need to shower after data collection, providing significant convenience for easy cleaning. The Emotiv Epoc X device offers users a 16-bit resolution with 128 SPS or 256 SPS options. The EmotivPRO (v3.5.3) application provided by Emotiv (EMOTIV Inc., San Francisco, CA, USA) records and manages the data collected with the device. The electrodes shown in Figure 3b are positioned according to the international 10/20 system [60]. Electrodes labeled F3, F4, AF3, AF4, F7, and F8 are used to monitor the participant’s neural activity. Electrodes T7, T8, FC5, and FC6 are positioned for auditory, visual, and speech functions, while P8 and P7 electrodes measure perception and numerical processing states. Electrodes placed at the back of the head, labeled O2 and O1, are for visual perception, response, and memory. CMS and DRL are ground electrodes [41].

### 3.2. Power Spectral Density (PSD)

EEG signals recorded from the scalp using surface electrodes are captured as voltage and time series data through computers. Understanding and analyzing these signals in terms of time and voltage is quite challenging. Therefore, it is necessary to convert the data from the time domain to the frequency domain. This study used the Welch power spectral density (PSD) method to transform the data into the frequency domain and identify frequency ranges.

In the Welch method, the length of the data is divided into *K* equal segments with an overlap. Each overlapping block has a length of *L*. The Welch estimate of the PSD is the average of the periodograms of the overlapping segments, as shown in Equation (1).

(1) $$P_{welch}(f)=\frac{1}{K.L.U}\sum_{i=0}^{K-1}{\left|\sum_{n=0}^{L-1}x_{i}\left(n\right).\omega \left(n\right)e^{-j2\pi fn}\right|}^{2}$$

(2) $$U=\frac{1}{L}\sum_{n=0}^{L-1}\omega^{2}(n)$$

In Equation (1), *K* represents the number of overlapping segments, *L* denotes their length, *N* is the length of the signal, and *f* is the frequency. In addition, $x_{i}\left(n\right)$ is the input signal, and ω(n) is the windowing function. In Equation (2), the value denoted by *U* represents the power of the window function used in Equation (1) [61].

### 3.3. Numerical Integration (Trapezoidal)

The present study used the trapezoidal numerical integration method to distinguish between data recorded with the LED (7 Hz) active and data recorded with the LED turned off. The trapezoidal rule is a commonly used method for calculating the area under curves in science and engineering applications. This method divides the integration data into small trapezoids and approximates the area to perform numerical integration. The general trapezoidal formula is given in Equation (3).

(3) $$\int_{a}^{b}f\left(x\right)dx=\frac{h}{2}\left[f\left(x_{0}\right)+2\sum_{i=1}^{n-1}f\left(x_{i}\right)+f(x_{n})\right]$$

where *a* and *b* represent the integration interval of the function, *h* denotes the width of each sub-trapezoid, and the points ($x_{0}$, $x_{1}$,...$\;x_{n}$) represent the equally spaced points within the interval.

### 3.4. Normalization (Z-Score)

Normalization is a scaling method that makes the analyzed data easier to process and distinguish in machine learning models. Different methods (e.g., min-max, L2-norm) are used for normalizing data. In this study, the Z-score method was used for normalization. The Z-score normalization process (Equation (4)) involves adjusting the data to have a mean of zero and a standard deviation of one:

(4) $$x^{\prime }=\frac{x-\mu }{\sigma }$$

where *x* represents the data to be normalized, μ is the mean of the data, and σ is the standard deviation of the data.

### 3.5. Analysis of Variance (ANOVA)

In the field of signal processing, features are extracted from datasets to distinguish between different classes. Since not all extracted features may be discriminative for the data, selecting or eliminating features may be necessary. In such cases where features are present, ANOVA is often used for selection and elimination. Most statistical software performs ANOVA on raw data [62]. ANOVA is a method used to test the variance differences between groups statistically. This method is expressed by Equation (5):

(5) $$SST=\sum_{i=1}^{k}\sum_{j=1}^{n_{i}}(Y_{ij}-\hat{Y}{)}^{2}$$

where $Y_{ij}$ represents the *j* observation in group *i*, $\hat{Y}$ denotes the mean of all observations, k indicates the number of groups, and $n_{i}$ represents the number of observations in group *i*.

### 3.6. Performance Parameters of BCI Systems

#### 3.6.1. Accuracy (Acc)

Accuracy is one of the most commonly used metrics in BCI systems. This metric is obtained by dividing the number of correctly identified examples by the total number of correctly and incorrectly identified examples. *Acc* is calculated using Equation (6).

(6) $$Acc=\frac{correctly\;classified\;predictions}{total\;number\;of\;examples}$$

In the studies conducted, accuracy is frequently calculated using hold-out methods. This approach divides the data into training and test sets. The system is trained with the training set, and a model is created. The created model is then tested with the test set. Typically, the data are shuffled and repeated, with the average of the obtained values being taken. This approach ensures the stability of the system [40].

#### 3.6.2. Information Transfer Rate (ITR)

ITR is a comprehensive parameter that provides information about the real-time usability of BCI systems. As an important performance metric, the ITR value is calculated using parameters such as the BCI system’s accuracy, the signal’s duration, and the number of classes. According to Shannon’s theory, ITR is expressed by Equation (7) [63].

(7) $${B_{t}=\log}_{2}K+p\log_{2}p+(1-p)\log_{2}(\frac{1-p}{K-1})$$

where *K* denotes the number of choices available to the system user, and *p* represents the system’s accuracy. The *ITR* value is calculated using $B_{t}$ (Equation (8)).

(8) $$ITR=60*\frac{Bt}{T}$$

where *T* represents the time allocated for a single prediction to be recognized by the system during the classification phase [46].

### 3.7. Classification Algorithms

#### 3.7.1. K-Nearest Neighbors (k-NN)

The k-NN algorithm is frequently used in BCI systems. This machine learning algorithm employs a simple approach where the class of an element to be classified is assigned based on the class of the nearest element to its value. The number of neighbors to be considered is determined by a pre-defined parameter *k*. Thus, the performance of the classifier is directly related to the value of *k*. Typically, *k* is chosen to be smaller than the square root of the total number of samples. The distance to the neighbors in k-NN is calculated using methods such as Euclidean (Equation (9)), Manhattan (Equation (10)), and Minkowski (Equation (11)) distances [64].

(9) $$d_{xy}=\sqrt{\sum_{i=1}^{k}{\left(x_{i-}y_{i}\right)}^{2}}$$

(10) $$d_{xy}=\sum_{i=1}^{k}⃒x_{i}-y_{i}\;⃒$$

(11) $$d_{xy}={\left(\sum_{i=1}^{k}{\left(\;⃒x_{i}-y_{i}\;⃒\right)}^{q}\right)}^{1/q}$$

Equations (9)–(11) represent *k* as the number of data points, *i* as the index of the data, and *d* as the distance.

#### 3.7.2. Support Vector Machine (SVM)

SVM is a widely used supervised learning method for classification tasks. This method aims to create a linear decision surface that provides the best separation between non-linearly separable data points. While some data can be linearly separated, other data cannot be. In such cases, kernel functions are used to map data into higher dimensions to achieve linear separation [65]. The study used the Gaussian radial basis function (RBF) kernel function. The Gaussian RBF is formulated as shown in Equation (12):

(12) $$K\left(x_{i,}x_{j}\right)=\varphi \left(x_{i}\right)\times \varphi \left(x_{j}\right)$$

where $x_{i}$ and $x_{j}$ are two data points. The vector weight of the decision surface created by the RBF kernel function is calculated using Equation (13).

(13) $$W=\sum_{i}a_{i}y_{i}\varphi \left(x_{i}\right)$$

The classifier balances the trade-off between dimensionality and flexibility by minimizing Equation (14) [65].

(14) $$\left[\frac{1}{n}\sum_{i=1}^{n}max\left(0.1-y_{i}\left(x^{k}x_{i}-b\right)\right)\right]+\lambda |w{|}^{2}$$

#### 3.7.3. Random Forest (RF)

The random forest (RF) classifier algorithm emerged as an alternative to boosting. As an extension of bagging [66], it offers advantages in terms of training time compared to other algorithms. The algorithm’s ease of application to parallel systems and high-dimensional data, along with its minimal number of parameters, are among its significant advantages over other classification methods. RF is a tree-based ensemble method where each tree relies on a randomly selected subset of variables. In this context, a random vector of real values with dimension $f\left(x\right)$ and an unknown common distribution for the randomly chosen target variable are considered. The main goal of the RF algorithm is to find a function that follows a loss function for the target variable and to minimize and smooth this function’s value [67].

#### 3.7.4. Linear Discriminant Analysis (LDA)

Linear discriminant analysis (LDA) is a commonly used machine-learning algorithm for distinguishing and classifying groups of attributes within a dataset. The algorithm aims to maximize the differences between attribute groups while minimizing the variations within each class [68]. In addition to classification, LDA is used for dimensionality reduction. The within-class and between-class scatter matrices of the algorithm are computed using Equation (15):

(15) $$S_{\omega }=\frac{\sum k\sum_{i\in c_{k}}\left(x^{i}-m_{k}\right)(x^{i}-m_{k}{)}^{T}}{N},\;S_{b}=\frac{\sum_{k}n_{k}(m_{k}-m)(m_{k}-m{)}^{T}}{N}$$

where $\frac{1}{n_{k}}\sum_{i\in c_{k}}x^{i}$ is used to calculate the mean value of class k. The expression $m=\frac{1}{N}\sum_{i}x^{i}$ represents the mean of the dataset. The algorithm can be differentiated by applying a Gaussian mixture model to the training data. The obtained models can be used to classify examples of the classes represented in the training data, although they are not suitable for new classes [69].

### 3.8. Participants

The research participants were aged between 18 and 45 years (avg. 30.7) and were selected from individuals who voluntarily agreed to participate and who had no health problems or dependencies (except smoking). The selected participants were thoroughly informed about the study before the experiments. The informed individuals were included in the study by filling out a volunteer consent form. The experiments were conducted with 10 participants, consisting of four females and six males. The study was conducted with the ethical approval of the Trabzon Kanuni Training and Research Hospital Medical Faculty, numbered 23618724.

### 3.9. Data Acquisition

**Figure 4:**
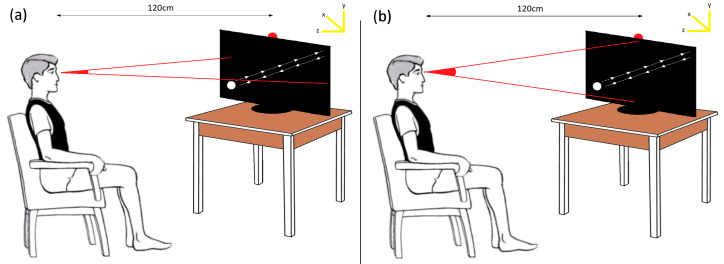
Representative maximum gaze angles formed along the subject’s X (**a**) and Y (**b**) axes.

The experimental phase was prepared by placing an LCD monitor with a refresh rate of 144 Hz, a response time of 1 ms, and a screen size of 27 inches (68 cm) on a flat table in an empty room. An LED with a frequency of 7 Hz was mounted at the center of the upper part of the screen. A chair was positioned perpendicular to the screen at a distance of 120 cm for the subject. The subject’s horizontal and vertical eye angles, relative to the distance from the screen, are depicted in visual representations in Figure 4a and Figure 4b, respectively.

The human eye has a curved structure with a visual field of 178° horizontally and 135° vertically. The limiting visual angle in the temporal direction is generally considered 105° [70]. An individual’s eye movements are crucial in eye movement-based systems, as more pronounced eye movements result in more meaningful recorded signals. However, excessively large eye angles indicate that the person is close to the visual target, which can be uncomfortable for the individual. In one study, individuals’ eye angles were 20–30° [71]. Table 2 presents the maximum eye movement angles resulting from the distance depicted in Figure 4 calculated for the X and Y axes.

In the human eye, there is an electrical potential called the corneoretinal potential (CRP) between the cornea and the retina. While the cornea has a positive electrical charge, the retina has a negative electrical charge. The CRP potential can be recorded as an EOG signal by placing electrodes around the eyes. Because EOG signals indicate eye position, they can be used to detect eye movements [72]. The angles between the four moving orbits in the study were determined by considering the angle changes occurring in the X and Y axes of the eye. While only the horizontal axis angles of the eye are used in the right and left movement orbit, only the vertical axis angles of the eye are used in the up and down movement orbit. In the right cross orbit, both the right and left axis angles of the eye are used together. In the left cross movement, the horizontal and vertical axis angles of the eye are used in the opposite direction to the right diagonal movement trajectory. Angular changes in the eye axis depending on the movement trajectories used are given in Table 3.

Many different movement trajectories were tested during the development of the current study. For example, signals were recorded for circular motion trajectory and triangular motion trajectory, and the recorded data were processed with the signal processing methods applied in the study. As a result of signal processing, it was seen that these two movements were not distinguishable by the BCI system since they had similar axis angles. As a result of the experiments, four trajectories that gave the best results were determined and used for the study. While trajectories with different axis motion angles can be classified by the system with high accuracy rates, it has been observed that trajectories with similar axis motion angles have a low separability rate.

After preparing the appropriate environment for the subject, the Emotiv Epoc X EEG device and the laptop to be used for data recording were set up in the room. The experimental room was isolated from external factors that could potentially distract the subject’s attention (e.g., noise and light).

**Figure 5:**
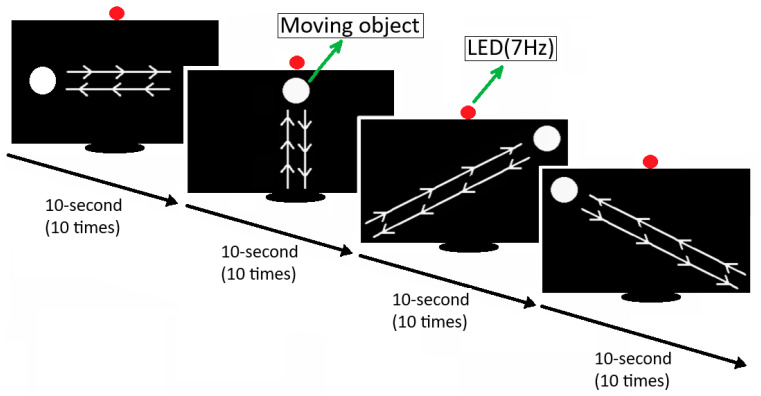
Stages of applying the approach.

Once the environment for data recording was prepared, participants were brought into the experimental room one by one. They were informed that the devices used in the study and none of the experimental phases posed any health risks. Then, they were asked to fill out a volunteer consent form. The next step was to fit the participant with the EEG device. The Emotiv Epoc X EEG device is wearable and can be applied using a saline solution. After fitting the participant with the EEG device using a saline solution, the quality of the electrode signals was checked via the EmotivBCI application provided by Emotiv, using the computer. Once the signal quality reached an adequate level (98%), the EEG headset was properly installed. The participant, now ready for the experiment, was instructed on the sequence in which to follow the white balls. The proposed approach involves four different movements for the participant to follow, moving right and left, moving up and down, moving across to the right, and moving across to the left. The sequence for applying the approach is exhibited in Figure 5.

**Figure 6:**
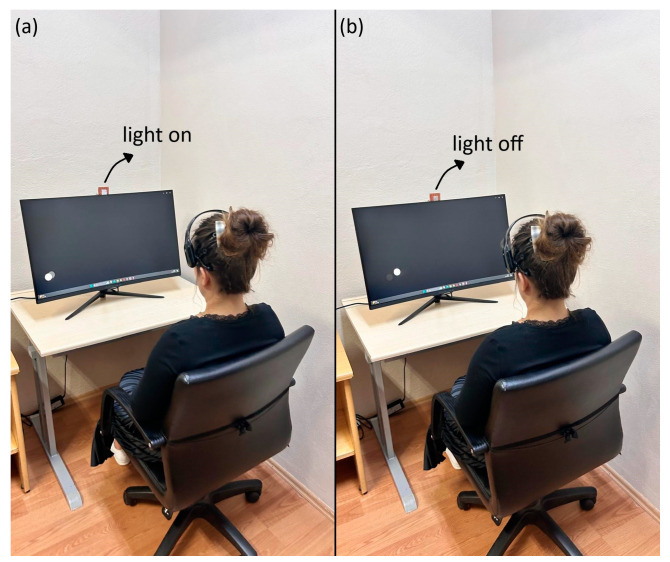
The EEG signal recording stage under light on (**a**) and light off (**b**) conditions.

In the initial data recording phase, an LED with a frequency value of 7 Hz was activated (Figure 6a). The experiment began with a 3 s beep sound for both right and left movements while the LED was active. A white marble, completing one full rotation per second, was displayed to the subject for 10 s. After 10 s, the data recording was completed with a 3 s beep sound. The experiment was repeated 10 times for both right and left movements in the same manner. A 1 min rest period was provided between each recording. Similarly, the experiment was conducted for up-down, right-cross, and left-cross movements, each with 10 repetitions. The phases of the experiment with the light on and off are illustrated in Figure 6.

In the second phase of data recording, the LED with a frequency of 7 Hz was turned off (Figure 6b). Initially, the subject was shown right and left movements for 10 s, accompanied by a 3 s initial beep sound. The recording was concluded with a 3 s beep sound. The experiment was repeated 10 times for both right and left movements in the same manner. Recordings were then made for other movements with 10 repetitions each, and the experiment was completed. In studies on eye movement-based BCIs [48,73,74], it was observed that channels AF3, F7, F8, and AF4 (located on the brain’s frontal lobe) are frequently used as active channels. In this study, these channels were considered likely to be effective.

**Figure 7:**
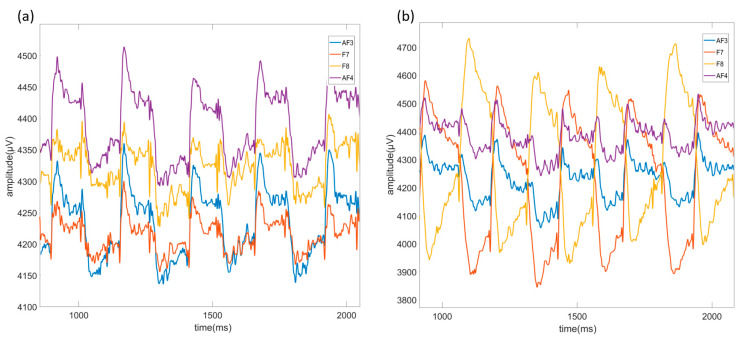
Up-down movement raw signals (**a**) and left-cross movement raw signals (**b**) of AF3, F7, F8, and AF4 channels caused by EOG artifacts in EEG signal.

**Figure 8:**
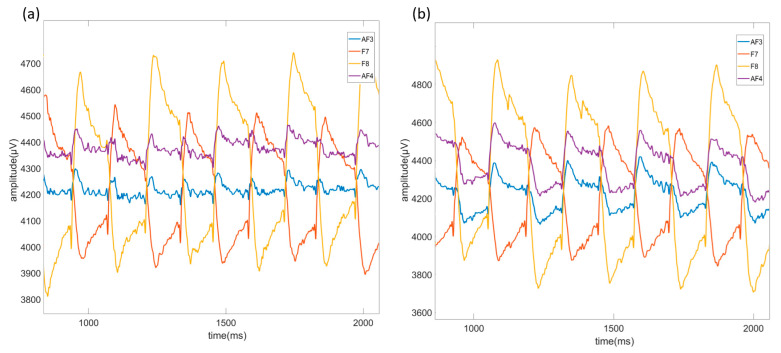
Right-left movement raw signals (**a**) and right-cross movement raw signals (**b**) of AF3, F7, F8, and AF4 channels caused by EOG artifacts in EEG signals.

Raw EEG data containing EOG artifacts recorded using AF3, F7, F8, and AF4 channels are shown in Figure 7a for up-down movement, Figure 7b for left-cross movement, Figure 8a for right-left movement, and Figure 8b for right-cross movement. Moreover, the data recorded with the light on are referred to as “illuminated data”, and the data recorded with the light off are referred to as “non-illuminated data”.

A total of 800 recordings were made throughout the experiments. The data were sampled at a frequency of 256 Hz. The recorded data were converted into .csv files using the EmotivPRO application from Emotiv. The converted data were then imported into the MATLAB (R2023b) environment for signal processing.

## 4. Results

**Figure 9:**
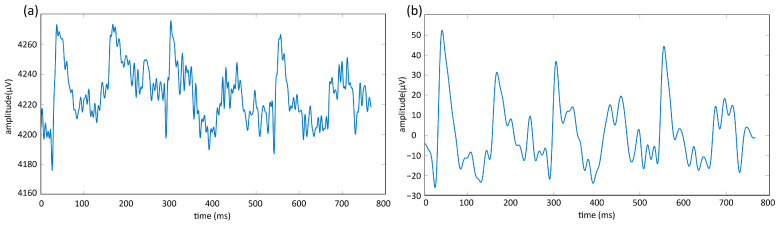
Unfiltered (**a**) and filtered (**b**) data examples for channel AF3.

In this study, 40 recordings were made for each subject with the light-on position, and 40 recordings were made with the light-off position during the data-recording phase. Overall, 80 recordings (each 16 s long) were obtained per subject using four different object routes and recordings consisting of 10 repetitions. All recorded data were transferred to the MATLAB environment, where the 3 s start and end beep sounds were removed. As a result, raw data with a dimension of 10 s (2560 × 14) were obtained. The raw data were segmented into 3 s segments (768 × 14) with 1 s overlaps. Consequently, five 3 s segments were obtained from each 10 s recording, resulting in 200 illuminated and 200 non-illuminated data segments per subject. The segmented 3 s illuminated and non-illuminated data were subjected to a fifth-order band-pass Butterworth filter in the 1–45 Hz range. The filtering process was performed using the filter command in MATLAB. An example of the unfiltered (a) and filtered (b) data from a randomly selected channel (AF3) is illustrated in Figure 9.

The filtered, illuminated, and non-illuminated data were analyzed using the Welch PSD method. Windowing was performed using the Hamming method, with a window length (WL) of 637 and an overlap value (noverlap) of 636, selected through trial and error. The Welch frequency resolution was used to determine the frequency ranges for both illuminated and non-illuminated data: 0–4 Hz (delta), 4–8 Hz (theta), 8–13 Hz (alpha), 13–30 Hz (beta), and 30–45 Hz (gamma). For each identified frequency band, features including kurtosis, mean, skewness, trapz, entropy, variance, mobility, and complexity were extracted, resulting in 40 features.

### 4.1. Channel Selection

**Figure 10:**
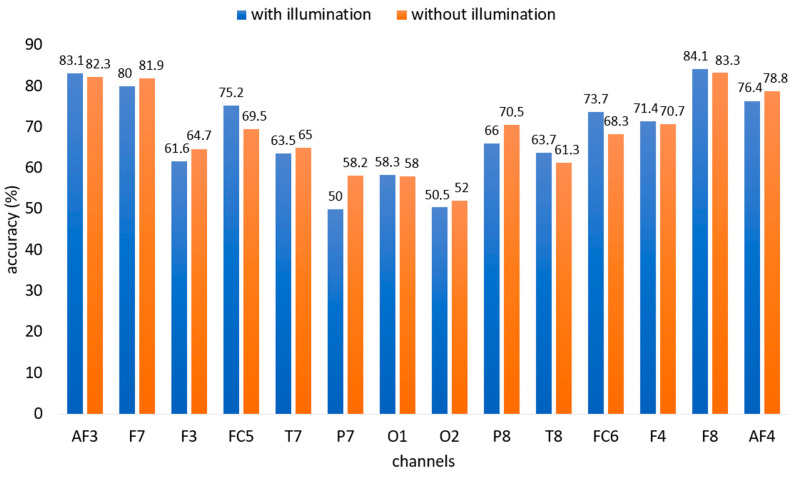
Classification accuracy rates of illuminated and non-illuminated data using all channels with an RF classifier.

Both illuminated and non-illuminated data were classified using RF, LDA, SVM, and k-NN classifiers across all channels to identify effective channels. The classification process was repeated 10 times, and the average accuracy values were computed. The RF classifier, which provided the highest accuracy for both illuminated and non-illuminated data, is presented in Figure 10.

As can be inferred from Figure 10, channels AF3, F7, F8, and AF4 exhibit the highest accuracy rates for both illuminated and non-illuminated data. These channels positioned around the eyes of the EEG device are effective for the study. Thus, these channels were selected as active channels for this study.

### 4.2. Eye Movement-Based BCI

**Figure 11:**
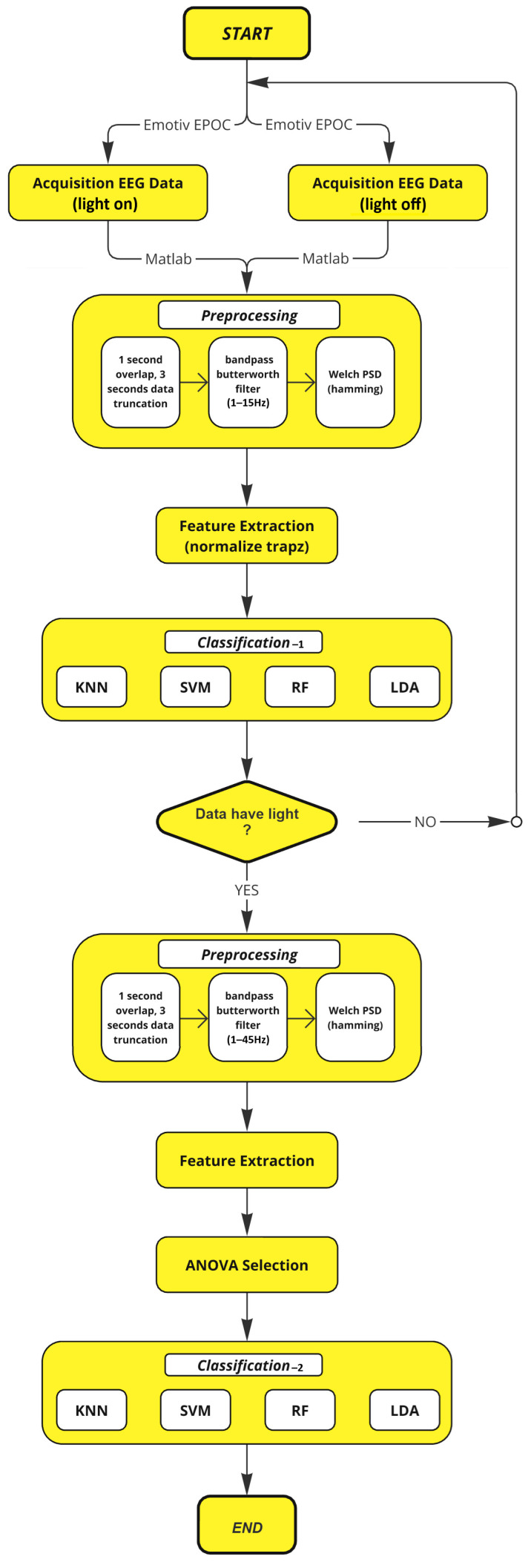
Algorithm of the designed BCI system.

Our BCI system consists of two sequential classification stages. In the first classification stage, the system differentiates between illuminated and non-illuminated data to identify the visual stimulus in the background. The illuminated data correctly predicted by the classifier in the first classification stage form the test set for the classification stage. The second classification stage is conducted to detect movements (left-right, up-down, right-cross, and left-cross) within the correctly predicted data. The algorithm of the designed BCI system is presented in Figure 11.

In the first classification stage (i.e., classification of illuminated and non-illuminated data), a fifth-order band-pass Butterworth filter with a 1–15 Hz range was applied to illuminated and non-illuminated data. The filtered data were then processed using the PSD method with a Hamming window of 637 sample length and 636 overlap, values chosen through trial and error. Using Welch frequency resolution, the frequency ranges of 1–10 Hz and 6–8 Hz were identified. Trapezoidal features were extracted from the identified 6–8 Hz and 1–10 Hz ranges using the *trapz* command in MATLAB. The two trapezoidal features obtained from the two frequency bands were normalized by calculating their ratio (Equation (16)) to yield a single normalized trapezoidal feature.

(16) $$z^{\prime }=\frac{trapz(\theta )}{trapz(\rho )}$$

where θ represents the trapezoidal value for the 6–8 Hz frequency range, while ρ denotes the trapezoidal value for the 1–10 Hz frequency range. The ratio of these two values was used to derive a single normalized trapezoidal feature.

**Figure 12:**
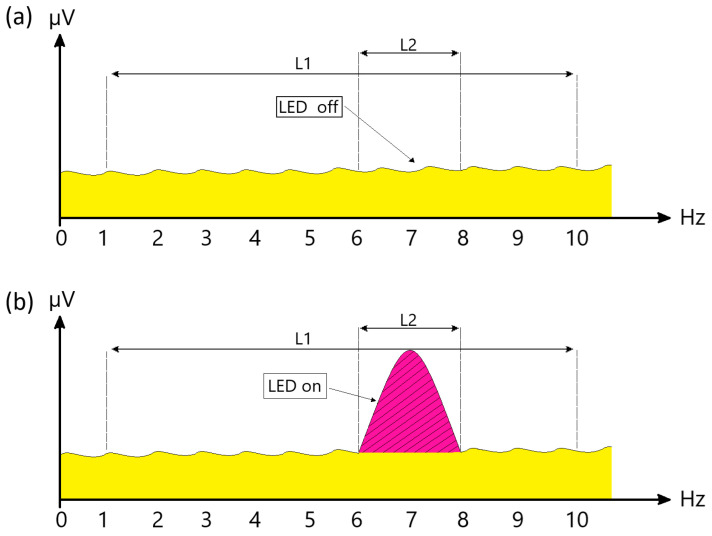
SSVEP potentials occurring in the LED off position (**a**) and LED on position (**b**).

In the designed BCI system, in light data, a potential pattern occurs in the 6–8 Hz band, since the LED is on in this range. Due to the formation of the pattern, the value of the normalized trapezoidal feature obtained as a result of the ratio for luminous data is greater than 1. In data without light, since the LED is off, no potential occurs in the 6–8 Hz range. Therefore, for data without light, the normalized trapezoidal feature ratio takes values close to 1. These trapezoidal features obtained in both cases are classified using machine learning algorithms. In Figure 12, the potentials occurring when the LED is off (a) and when the LED is on (b) are shown representatively.

When looking at Figure 12a (LED off), it is observed that there is no potential change in the 6–8 Hz range. In contrast, in Figure 12b (LED on), a potential change in the 6–8 Hz range is evident. Whether the data is illuminated or non-illuminated is determined according to the size of the trapezoidal value obtained by the proportion of the L1 and L2 distances, which represent the frequency range of the potentials occurring. Thus, the presence or absence of light in the area where the system user is looking is decided by the BCI system. In cases where light cannot be detected, the system does not activate, and the system user is provided with the opportunity to use a safe BCI system.

**Figure 13:**
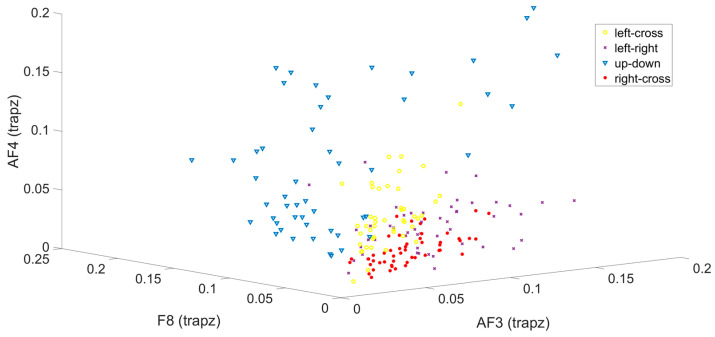
3D feature scatter plot created using channels AF3, F8, and AF4.

The three-dimensional feature scatter plot created using the selected effective channels AF3, F8, and AF4 is illustrated in Figure 13.

In the first classification stage, a single feature was extracted; however, four features were derived by combining the data obtained from the selected effective channels (AF3, F7, F8, and AF4). The feature data were partitioned into 75% training and 25% test sets using the holdout method to classify between illuminated and non-illuminated data. In order to test the robustness of the system, training and test data of a randomly selected subject (subject-2) were limited to training data using machine learning algorithms. The accuracy rates obtained are shown in Table 4.

When Table 4 is examined, the accuracy rates obtained are within acceptable ranges, there is no under-fitting or over-fitting, and the number of trials is sufficient to demonstrate the method’s robustness. After examining the robustness of the system, classification was first performed using RF, LDA, k-NN, and SVM classifiers. The classification processes were repeated 10 times, and the average results were computed to determine the accuracy rate for each subject. The accuracy rate results of the first classification stage are shown in Table 5. In Table 5, the illuminated data are given as class 1, and the non-illuminated data are given as class 2.

According to Table 5, accuracy rates of 99.57%, 99.11%, 95.74%, and 98.22% were achieved for the RF, SVM, LDA, and k-NN algorithms, respectively. It was observed that the RF and SVM algorithms yielded relatively better results compared to the other methods. The classification results provided by these algorithms indicate that the approach successfully detects the visual stimulus used for safety purposes in the background system. In the first classification stage, the illuminated data correctly predicted by the classifiers served as the test data for the second classification stage.

**Figure 14:**
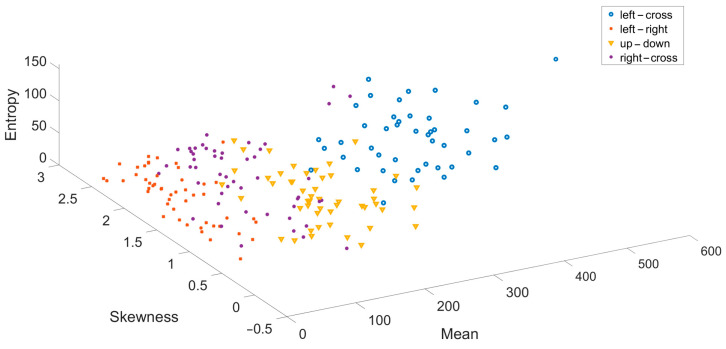
3D scatter plot of entropy, skewness, and mean features for illuminated data from channel AF3.

In the second classification stage, all accurately predicted illuminated data were processed using a fifth-order Butterworth filter within the 1–45 Hz range. The filtering process utilized the filtfilt function in MATLAB. Following the filtering, the Welch PSD method was applied to the filtered data using a Hamming window with a window length of 637 and an overlap value of 636. Using Welch frequency resolution, the frequency ranges of 0–4 Hz (delta), 4–8 Hz (theta), 8–13 Hz (alpha), 13–30 Hz (beta), and 30–45 Hz (gamma) were identified. For each identified frequency band, features including kurtosis, mean, skewness, trapz, entropy, variance, mobility, and complexity were extracted. As an example, Figure 14 presents a three-dimensional feature scatter plot created using randomly selected entropy, mean, and skewness features from the randomly selected channel AF3.

Since eight features were extracted for each frequency band, 40 features were obtained for all frequency bands. Combining the data from the identified active channels increased the number of features to 160. The feature data were divided into 75% training and 25% test sets using the holdout method. The correctly predicted data from the first classification stage were identified from the test set, while incorrectly predicted data were excluded from the test set. The data were classified using RF, LDA, k-NN, and SVM classifiers. The classification was repeated 10 times, and each subject’s accuracy rates and ITR values were calculated by averaging the results. The accuracy and ITR rates obtained from the classification are provided in Table 6. Table 6 shows up-down movement as class 1, right-left movement as class 2, left-cross movement as class 3, and right-cross movement as class 4.

Table 6 reveals accuracy rates of 97.89%, 97.37%, 95.12%, and 90.39% and ITR values of 36.75, 36.06, 33.4, and 28.01 (bits/min) for the RF, SVM, LDA, and k-NN algorithms, respectively. The RF classifier provided the best results, while the SVM classifier yielded very close results. In comparison, LDA and k-NN performed relatively lower than the other classifiers. In the classification process for distinguishing illuminated data in the second stage, the RF classifier demonstrated the best performance with an accuracy rate of 97.89% and an ITR value of 36.75 bits/min.

### 4.3. Future Selection

**Figure 15:**
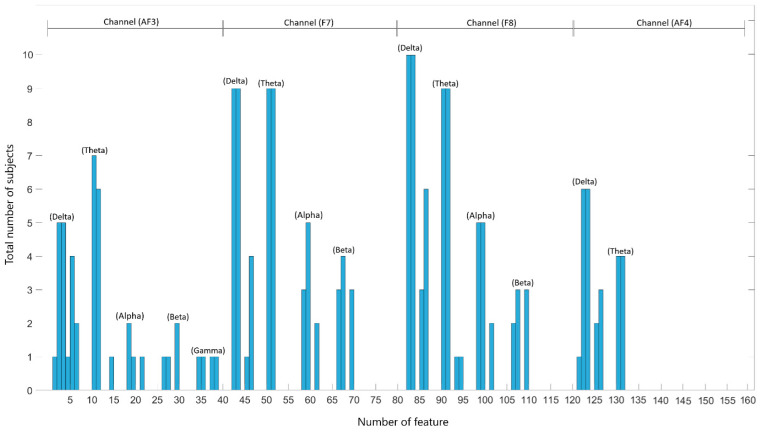
When the 160 features are ranked according to their ANOVA scores, the number of occurrences of the top 20 features with the highest ANOVA scores in the participants.

**Figure A1:**
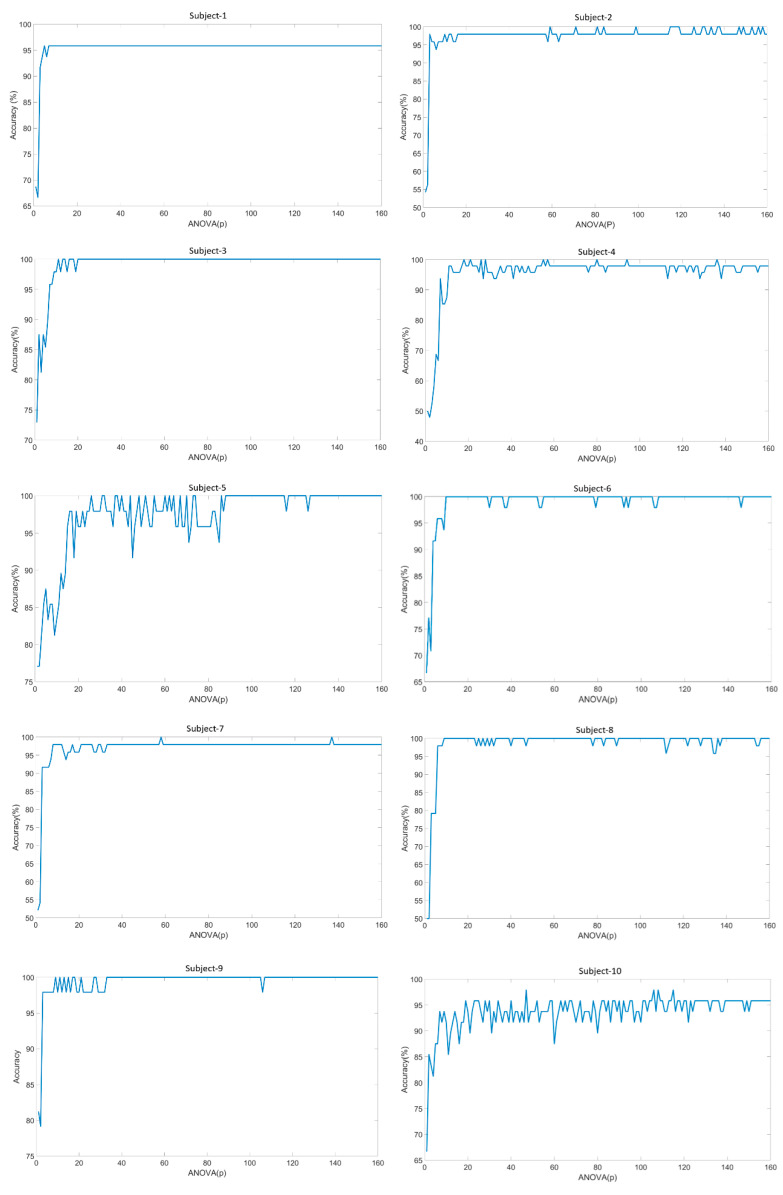
Stepwise ANOVA score accuracy rates for all subjects.

For the classification of illuminated data, feature selection was performed using the ANOVA method among the 160 features. The best features were identified for each subject. The classification was then performed using the RF algorithm, starting from the best feature and including the next one iteratively. Accuracy rate graphs based on the number of features for each subject are provided at the end of the study (Figure A1). The graph for the top 20 features for each subject is depicted in Figure 15.

Figure 15 shows that the features numbered 82 and 83 from channel F8 (i.e., the mean and skewness parameters of the delta band) were identified as the best features for all participants. Additionally, the features numbered 92 and 93 from channel F8, corresponding to the theta band’s trapezoidal and entropy features, are among the top 20 features for nine participants. Moreover, features 42, 43, 52, and 53 from channel F7, corresponding to the mean and skewness of the delta band and the trapezoidal and entropy of the theta band, were identified as the best features for nine participants. In conclusion, the study indicates that the delta band is the most dominant, with the best features being mean and skewness. The trapezoidal and entropy features and the theta band are also prominent after the delta band.

**Figure 16:**
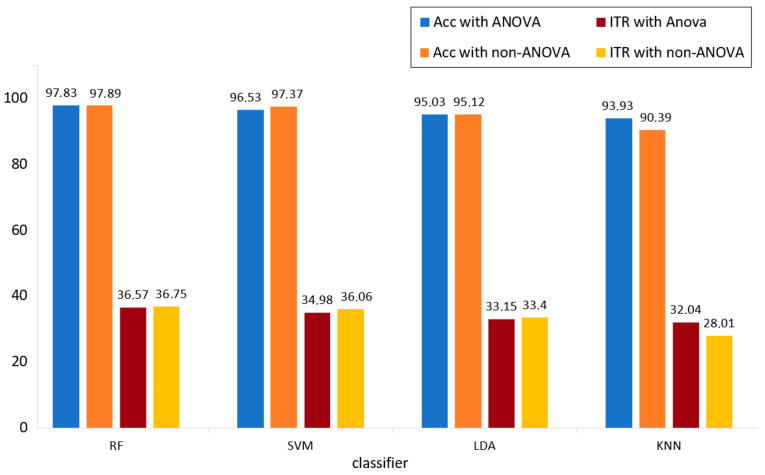
Comparison of the results of the illuminated (moving objects) classification stage before and after ANOVA accuracy rate and ITR value.

A new feature set was created using the mean and skewness features from the delta band and the trapezoidal and entropy features from the theta band, as identified through the ANOVA feature selection method. Since each channel contains four features, the total feature set consists of 16 features. The second classification stage was repeated using the new feature set. Accuracy rates and ITR values obtained from classification results are presented in Table 7 and Figure 16. In Table 7, up-down movement is given as class 1, right-left movement as class 2, left-cross movement as class 3, and right-cross movement as class 4.

Examining Table 7 and Figure 16 indicates that the accuracy rate for the k-NN classifier significantly increased to 93.33% from 90.39%, with the ITR value rising to 32.04 (bits/min) from 28.01 (bits/min). For the RF and LDA classifiers, very similar values were obtained before and after ANOVA. However, the SVM classifier’s accuracy rate decreased to 96.53%, and the ITR value declined to 36.06 bits/min. As a result, the number of features was reduced from 160 to 16. The ANOVA feature selection method was found to be effective for the current study.

General performance values of the system including classification-1 and classification-2 stages are given in Table 8. The overall accuracy rate of the system was calculated by dividing the number of correctly predicted illuminated data in the second classification stage by the total number of illuminated data found in the first classification stage. When Table 7 is reviewed, it is seen that the designed BCI system gives the best performance values with the RF classifier with an accuracy rate of 97.42% and an ITR value of 35.75 (bits/min).

## 5. Discussion and Future Work

When the studies in the field are examined, they show that a significant part of the research in VEP-based BCI systems focuses on system performance [75]. However, the visual stimuli used in these systems seriously threaten the eye health of users, reduce system comfort, and reduce system usage time [25,26]. In fact, in some studies, it was observed that the subjects could not complete the experimental stages due to the negative effects of visual stimuli on the users [44]. VEP-based BCI systems are also unsuitable for individuals with inadequate eye health, as these systems require the ability to perceive the frequency values of visual stimuli. Additionally, while EEG devices used in these systems are more cost-effective compared to many EEG recording methods (such as MEG and fMRI), they are still not sufficiently affordable to be widely adopted. Moreover, these devices are inadequate in terms of usability and comfort [7].

This study proposed an innovative approach based on the hybrid method to minimize the negative effects of visual stimuli contained in VEP-based BCI systems on the user. Instead of visual stimuli, four different moving objects, each moving in different trajectories, were used in the approach. Classification of moving objects was carried out using EOG artifacts found in the recorded EEG signals. However, the system may be activated unintentionally when the user makes the same eye movement as moving objects. This problem is also seen in many other eye movement-based BCI studies [47,48,49]. Although various actions (for example, serial blinking or looking in a certain direction) have been tried to solve the problem, these methods complicate the use of the system and make it difficult for users to focus, increasing error rates. As a solution to this problem in the developed system, the user can activate the system by using the 7 Hz LED placed on the top of the computer screen, without being exposed to the negative effects of any visual stimulus and without focusing on the stimulus. The system checks the presence of the background LED via the SSVEP method when EEG data is received. Detection of the LED indicates that the user made a conscious eye movement to activate the system. Otherwise, when the LED is not detected, it is concluded that the eye movements are independent of the system and the system should not be active. Since there is only one LED, the stimulating brightness of the system is low, and the user does not have to focus directly on the LED. Thus, the system prevents users from experiencing a visual flash sensation, allowing users to safely use the system with moving objects without focusing on any visual stimuli.

In summary, in the study, while the control of moving objects was done using EOG artifacts contained in EEG signals, the LED placed in the background was detected using the SSVEP method. The system’s classification of moving objects using EOG artifacts has been proposed as an alternative to visual stimuli in visual stimulus-based BCI systems. Thus, it is aimed to find solutions to the problems of the feeling of glare in the eyes caused by visual stimuli, the negative impact on system comfort and usability due to the feeling of glare, eye disorders caused by long-term use, and reducing the system usage time. During the commissioning phase of the system using the LED in the background, the SSVEP method aims to enable the system to be safely activated and deactivated by the user. The success rate of the system during its activation phase is important because it is not difficult to foresee that systems that make wrong decisions will cause difficulties for paralyzed individuals. Thus, constant blinking, etc., is required to activate the system in the field. An alternative solution to these methods has also been provided.

Table 9 provides basic information on some visual stimulus-based studies using the Emotiv Epoc EEG device.

As can be inferred from previous studies [50,76,79], the number of subjects is relatively insufficient compared to the general standard. The number of subjects directly affects the performance metrics of the systems. Compared to other studies, the number of subjects used in this study is moderate. The accuracy rate is among the most critical parameters for the usability of systems. As shown in Table 8, the accuracy rate obtained in this study can be considered effective. Additionally, some studies [77,79,80,81] report that the ITR values are either not computed or are low. The ITR value reflects the speed of the system and is the most important parameter for assessing its practical applicability. The ITR value of 36.7 bits/min achieved in this study is considered successful compared to the values considered in the studies reviewed.

During the experiments, illuminated data were recorded without requiring any focus on visual stimuli or eye contact. Nevertheless, visual stimuli still caused discomfort for the subjects over time. Although this study significantly alleviates the reliance on visual stimuli for the system user, visual stimuli can still cause discomfort in various ways. Furthermore, the Emotiv Epoc X EEG device has its own disadvantages. These drawbacks include the excessive pressure it applies to the user’s head during use exceeding 30 min, the inability to adjust the device to fit different head sizes, electrode oxidation, and continuous drying. Furthermore, the device’s sensor contact points cannot be altered, leading to variability in contact points for individuals with different head sizes. Despite these issues, the device is preferred considering its advantages, such as affordability, portability, long battery life, ease of use and setup, and no need for any cleaning after use.

In BCI systems, using fewer EEG channels makes the system more efficient. According to the literature [76,80], researchers often increase the number of channels to enhance accuracy rates. In contrast, the current study utilized only channels F7, AF3, AF4, and F8 of the Emotiv Epoc X EEG device. Future research could further reduce the number of channels by evaluating the performance of effective waveforms for each channel.

In VEP-based BCI systems, brightness (lux) is an important parameter because the amount of light reaching the eye from the source creates a potential change in the brain. Studies on the intensity of light on SSVEP have been carried out in the field. The majority of research has explored illumination levels under 30 lx, revealing that as illumination intensity rises, the SSVEP response tends to improve [85,86,87,88]. However, a study showed that the highest illumination value used during the experiments did not give the best results [58]. Moreover, some studies have shown that higher brightness changes also lead to greater discomfort [86,88]. In this study, by reducing the number of visual stimuli to 1, the amount of light the user is exposed to is reduced and the need to focus on the stimulus is eliminated. However, no investigation has been made about the brightness of the visual stimulus used. System performance can be increased by increasing the brightness level of the stimulus, or a more comfortable system can be designed by decreasing it. Another issue is that the monitor used to display the stimuli emits light and is annoying and unusable because it has to be constantly in front of the patients. Moreover, almost all studies reviewed consist of single-session or daily recordings. However, systems should be capable of producing consistent responses for the same user on different days. In summary, the model created from data recorded on the first day for person A should be compatible with data recorded on the second day for the same person. Another problem is that the designed system has not been tested in real time. Future research will address real-time implementation of the system and other issues identified.

## 6. Conclusions

This study proposes a hybrid BCI system that uses SSVEP and EOG methods to minimize the negative effects of visual stimulus-based BCI systems on the user. Visual stimuli can be quite detrimental to the user’s eye health, as they require direct focus during system use. Additionally, this negatively affects system comfort and reduces usage time. The study proposes an innovative approach that uses white balls moving in different trajectories instead of visual stimuli to provide solutions to the mentioned problems. The system produces EOG artifacts potentials within EEG signals using white balls, and moving objects are classified using these EOG artifacts. However, the study also acknowledges the major drawback of the proposed approach. Since such systems are based on eye movements, they can also remain active in the user’s independent movements. In the study, this problem is solved through the SSVEP method by using a single 7 Hz frequency LED with a low brightness level, which is placed in the upper middle part of the screen, where the user does not need to focus. The purpose of using the LED is to detect whether the system user is looking at the screen containing moving objects. When the user looks at the screen, SSVEP is triggered, and the system is activated. If the screen is not looked at, the system is not triggered and is disabled. This ensures that the user can use the system safely.

In the study, a two-stage classification process was applied using 10 healthy subjects. In the first classification stage, SSVEP was triggered via LED, and whether the LED was active or not was verified. While the system does not react when the LED is not active, when it is active, the second classification stage is started. In the second classification stage, recorded EOG signals were classified using machine learning algorithms. As a result of the experiments, the RF machine learning algorithm achieved an accuracy rate of 99.57% for the first classification stage and 97.83% for the second classification and an ITR value of 36.75 bits/min. Considering the overall performance of the system, including classification-1 and classification-2 stages, the RF machine learning algorithm gave the best result with an accuracy rate of 97.42% and an ITR value of 35.75 bits/min. Additionally, effective channels and wavebands for the proposed system were identified in the study. The proposed hybrid BCI system eliminates the need for focusing on visual stimuli, reducing the number of visual stimuli in VEP-based BCI systems to a minimum and manageable level. Additionally, the study offers an innovative perspective to the field by proving that visual stimulus-based BCI systems can be used in a different way by using moving balls, without requiring direct focusing on the LED.

## Figures and Tables

**Table 1:** Characteristics of EEG wave bands.

Wave	Frequency Range (Hz)	Amplitude Range (μV)
Delta (δ)	0.5–4	1–120
Theta (θ)	4–7	20–100
Alfa (α)	7–12	30–50
Beta (β)	12–30	5–30
Gamma (γ)	30+	variable

**Table 2:** Maximum eye angles of the subject on the X-Y axes as a function of Z distance.

Axis	X	Y	Z
Distance (cm)	61	34	120
Gaze Angle (°)	28.5	16.1	-

**Table 3:** Angle changes caused by movement trajectories in the eye axes.

Trajectory	*X* Axis		*Y* Axis	
Right-left	right	left	right	left
	+14.2	−14.2	0	0
Up-down	up	down	up	down
	0	0	+8.1	−8.1
Right-cross	top right	bottom left	top right	bottom left
	+14.2	−14.2	+8.1	−8.1
Left-cross	top left	bottom right	top left	bottom right
	−14.2	+14.2	+8.1	−8.1

**Table 4:** Classification accuracy rate results of holdout test and training data of a randomly selected subject (subject-2) to measure the robustness of the system.

Classifier	Holdout Data	Trial-1	Trial-2	Trial-3	Trial-4	Trial-5	Trial-6	Trial-7	Trial-8	Trial-9	Trial-10	Avg. (%)
SVM	train	100.0	99.34	100.0	100.0	100.0	100.0	99.34	100.0	100.0	99.34	99.80
	test	100.0	100.0	97.92	97.92	97.92	100.0	97.92	97.92	100.0	97.92	98.75
k-NN	train	100.0	98.02	95.83	98.02	98.02	98.02	100.0	100.0	100.0	99.34	98.72
	test	95.83	89.58	91.67	93.75	93.75	93.75	93.75	95.83	95.83	97.92	94.16
LDA	train	98.68	99.34	99.34	100.0	99.34	99.34	98.68	99.34	100.0	99.34	99.34
	test	97.92	100.0	100.0	100.0	100.0	97.92	97.92	97.92	97.92	97.92	98.75
RF	train	99.34	100.0	100.0	100.0	100.0	100.0	100.0	100.0	100.0	100.0	99.93
	test	100.0	100.0	95.83	100.0	95.83	97.92	97.92	97.92	100.0	100.0	98.54

**Table 5:** Classification accuracy rates of illuminated and non-illuminated data using all features for channels AF3, F7, F8, and AF4.

Classifier	Classes	Sub-1	Sub-2	Sub-3	Sub-4	Sub-5	Sub-6	Sub-7	Sub-8	Sub-9	Sub-10	Avg. (%)
SVM	Class 1	100.0	100.0	99.37	100.0	99.68	97.50	100.0	100.0	100.0	99.37	99.59
	Class 2	97.08	100.0	100.0	100.0	100.0	100.0	95.20	95.41	98.75	100.0	98.64
	Acc.	98.54	100.0	99.68	100.0	99.37	98.75	97.60	97.70	99.37	99.68	99.11
k-NN	Class 1	100.0	100.0	98.95	100.0	98.95	100.0	100.0	98.95	100.0	98.95	99.58
	Class 2	96.45	98.95	100.0	99.79	100.0	96.45	98.95	100.0	99.79	100.0	99.03
	Acc.	98.22	99.47	99.79	99.89	99.47	98.22	99.47	99.79	99.89	99.47	99.30
LDA	Class 1	96.12	97.50	99.11	100.0	100.0	99.37	100.0	98.95	98.95	100.0	99.00
	Class 2	92.00	91.66	94.37	96.04	89.58	92.29	91.45	89.37	95.20	92.91	92.48
	Acc.	94.06	94.58	96.74	98.02	94.79	95.83	95.72	94.16	97.07	96.45	95.74
RF	Class 1	100.0	99.37	100.0	99.79	100.0	99.68	99.79	98.95	100.0	98.95	99.65
	Class 2	100.0	100.0	98.54	100.0	99.37	98.75	100.0	100.0	98.33	100.0	99.49
	Acc.	100.0	99.68	99.27	99.89	99.68	99.21	99.89	99.47	99.16	99.47	99.57

Class 1: illuminated data, class 2: non-illuminated data.

**Table 6:** Illuminated data classification accuracy results using channels AF3, F7, F8, and AF4.

Classifier	Classes	Sub-1	Sub-2	Sub-3	Sub-4	Sub-5	Sub-6	Sub-7	Sub-8	Sub-9	Sub-10	Avg. (%)
SVM	Class 1	93.33	100.0	97.50	95.83	97.50	94.16	99.16	100.0	97.50	97.50	97.24
	Class 2	100.0	100.0	92.50	99.16	95.00	91.66	100.0	98.33	100.0	90.00	96.66
	Class 3	100.0	100.0	100.0	100.0	99.16	100.0	100.0	100.0	100.0	100.0	99.91
	Class 4	91.66	98.33	95.00	93.33	95.00	99.16	95.00	97.50	100.0	91.66	95.66
	Acc.	96.25	99.58	96.25	97.08	96.66	96.25	98.54	98.95	99.37	94.79	97.37
	ITR	34.44	39.28	34.46	35.52	35.04	34.58	37.66	38.20	38.92	32.58	36.06
k-NN	Class 1	89.58	98.33	83.33	100.0	91.67	91.67	91.67	70.83	78.33	98.33	89.37
	Class 2	93.64	90.83	91.67	91.67	83.33	75.00	83.33	100.0	91.25	75.83	87.65
	Class 3	100.0	100.0	100.0	100.0	100.0	100.0	100.0	100.0	100.0	100.0	100.0
	Class 4	95.83	87.50	83.33	75.00	75.00	91.67	91.67	83.33	77.08	85.00	84.54
	Acc.	94.76	94.16	89.58	91.67	87.50	89.58	91.67	88.54	86.66	89.79	90.39
	ITR	32.54	32.04	27.05	29.08	25.46	27.05	29.08	26.10	24.43	27.25	28.01
LDA	Class 1	83.33	100.0	100.0	95.00	100.0	89.16	90.0	96.66	100.0	94.16	94.83
	Class 2	93.33	100.0	91.66	92.50	96.66	83.33	95.83	97.50	98.33	70.00	91.91
	Class 3	100.0	100.0	100.0	100.0	100.0	100.0	100.0	100.0	100.0	100.0	100.0
	Class 4	90.00	95.00	100.0	95.00	81.66	95.83	95.83	97.50	91.66	95.00	93.74
	Acc.	91.66	98.75	97.91	95.62	94.58	92.08	95.41	97.91	97.50	89.79	95.12
	ITR	29.32	37.93	36.58	33.98	32.29	29.97	33.44	36.78	36.18	27.59	33.40
RF	Class 1	95.83	100.0	100.0	98.33	97.50	95.00	98.33	97.50	100.0	96.66	97.91
	Class 2	100.0	100.0	97.50	98.33	98.33	93.33	98.33	96.66	98.33	95.00	97.58
	Class 3	100.0	100.0	100.0	100.0	100.0	100.0	100.0	100.0	100.0	100.0	100.0
	Class 4	93.33	95.83	98.33	96.66	96.66	91.66	95.00	99.16	99.16	95.00	96.07
	Acc.	97.29	98.96	98.95	98.33	98.12	95.00	97.91	98.33	99.37	96.66	97.89
	ITR	35.88	38.20	38.20	37.30	37.06	32.91	36.78	37.42	39.00	34.80	36.75

Class 1: up-down movement, class 2: right-left movement, class 3: left-cross movement, class 4: right-cross movement.

**Table 7:** Classification results of features obtained from illuminated data using ANOVA with channels AF3, F7, F8, and AF4.

Classifier	Classes	Sub-1	Sub-2	Sub-3	Sub-4	Sub-5	Sub-6	Sub-7	Sub-8	Sub-9	Sub-10	Avg. (%)
SVM	Class 1	93.33	98.33	93.33	90.0	94.16	95.0	99.16	96.66	100.0	99.16	95.91
	Class 2	100.0	100.0	85.83	96.66	96.66	91.66	96.66	94.16	99.16	90.0	95.07
	Class 3	100.0	100.0	100.0	100.0	99.16	100.0	100.0	100.0	100.0	100.0	99.91
	Class 4	95.0	96.66	93.33	93.33	98.33	95.0	97.5	96.66	99.16	87.5	95.24
	Acc (%)	97.08	98.75	93.12	95.0	97.08	95.41	98.33	96.87	99.58	94.16	96.53
	ITR	35.44	37.93	30.79	32.91	35.79	33.43	37.21	35.25	39.28	31.81	34.98
k-NN	Class 1	97.5	100.0	96.66	97.5	91.66	92.5	90.0	93.33	92.5	99.16	95.08
	Class 2	79.16	99.16	91.66	99.16	91.66	80.0	87.5	90.0	95.83	85.83	89.99
	Class 3	100.0	100.0	100.0	100.0	100.0	100.0	100.0	100.0	100.0	100.0	100.0
	Class 4	90.0	95.83	95.83	95.83	73.33	91.66	90.0	90.83	96.66	86.66	90.66
	Acc (%)	91.66	98.75	96.04	98.12	89.16	91.04	91.87	93.54	96.25	92.91	93.93
	ITR	29.35	37.93	34.10	37.20	26.84	28.73	29.85	31.33	34.43	30.69	32.04
LDA	Class 1	86.66	97.5	98.33	92.5	95.83	90.0	93.33	90.0	99.16	91.66	93.49
	Class 2	95.83	98.33	85.0	95.0	99.16	86.66	97.5	92.5	96.66	83.33	92.99
	Class 3	100.0	100.0	100.0	100.0	100.0	100.0	100.0	100.0	100.0	100.0	100.0
	Class 4	88.33	99.16	85.83	92.5	86.66	90.0	95.83	98.33	100.0	100.0	93.66
	Acc (%)	92.70	98.75	92.29	95.0	95.41	91.66	96.66	95.20	98.95	93.75	95.03
	ITR	30.39	37.93	30.11	32.84	33.50	29.18	35.04	33.07	38.29	31.27	33.15
RF	Class 1	93.33	98.33	98.33	94.16	98.33	94.16	99.16	95.83	100.0	95.0	96.66
	Class 2	100.0	100.0	95.83	100.0	97.5	96.66	95.83	99.16	100.0	98.33	98.33
	Class 3	100.0	100.0	100.0	100.0	100.0	100.0	100.0	100.0	100.0	100.0	100.0
	Class 4	93.33	97.5	96.66	95.83	94.16	96.66	97.5	96.66	100.0	95.0	96.33
	Acc (%)	96.66	98.95	97.70	97.5	97.5	96.87	98.12	97.91	100.0	97.08	97.83
	ITR	35.01	38.20	36.22	36.04	36.15	35.28	36.94	36.58	40.0	35.35	36.57

Class 1: up-down movement, class 2: right-left movement, class 3: left-cross movement, class 4: right-cross movement.

**Table 8:** General performance table of the system including all classification stages.

Classifier	Parameter	Sub-1	Sub-2	Sub-3	Sub-4	Sub-5	Sub-6	Sub-7	Sub-8	Sub-9	Sub-10	Avg.
SVM	Acc.	95.63	98.75	92.80	95.0	96.46	94.20	95.94	94.64	98.95	93.85	95.62
	ITR	33.43	37.66	30.25	32.68	34.46	31.77	33.81	32.27	37.98	31.38	33.56
k-NN	Acc.	90.00	98.22	95.83	98.01	88.68	89.41	91.38	93.34	96.14	92.41	93.34
	ITR	27.45	36.85	33.67	36.55	26.22	26.89	28.79	30.82	34.05	29.84	31.11
LDA	Acc.	87.19	93.39	89.28	93.11	90.43	78.95	87.68	85.37	91.62	88.07	88.49
	ITR	24.89	30.88	26.77	30.57	27.86	18.47	25.32	23.32	29.03	25.67	26.27
RF	Acc.	96.66	98.63	96.98	97.39	97.18	96.10	98.01	97.39	99.16	96.56	97.42
	ITR	34.71	37.47	35.13	35.68	35.40	34.01	36.55	35.68	38.33	34.58	35.75

**Table 9:** Comparison of the current study with previous studies using the Emotiv Epoc EEG device.

Ref.	Year	Method	Datasets	Number of Participants	Acc (%)	ITR (bits/min)
[76]	2024	SVM	Own data	3	90.1	27.3
[77]	2024	CNN	Own data	16	97.6	-
[78]	2024	SVM	Own data	28	88.4	27.7
[79]	2023	R-CNN	Own data	4	84.0	15.8
[80]	2023	CNN-LSTM	Guinea-Bissau and Nigeria epilepsy	-	92.5	-
[81]	2023	k-NN	arXiv [82]	23	89.5	-
[83]	2023	CNN	Own data	25	75.1	17.1
[84]	2022	SVM	Own data	15	91.0	28.4
[50]	2021	LSTM	Own data	5	96.9	40.3
Our Work	2024	RF	Our data	10	97.9	36.7

## Data Availability

Data used in this study were recorded at Gumushane University Vocational School with ethical approval number 23618724 from the Trabzon Kanuni Training and Research Hospital Medical Faculty. Upon request, the data may be shared with the requesting party by the corresponding author.
